# Comparison of Au Nanoparticle/Poly(9-vinylcarbazole) Thin-Film Electrogeneration at 3 Distinct Liquid/Liquid Interfaces: Water/1,2-Dichloroethane, /α,α,α-Trifluorotoluene, Or/Ionic Liquid

**DOI:** 10.1021/acs.langmuir.4c03265

**Published:** 2024-11-05

**Authors:** Leila Nazari, Talia Jane Stockmann

**Affiliations:** Department of Chemistry, Core Science Facility, Memorial University of Newfoundland, 45 Artic Avenue, St. John’s, Newfoundland and Labrador A1C 5S7, Canada

## Abstract

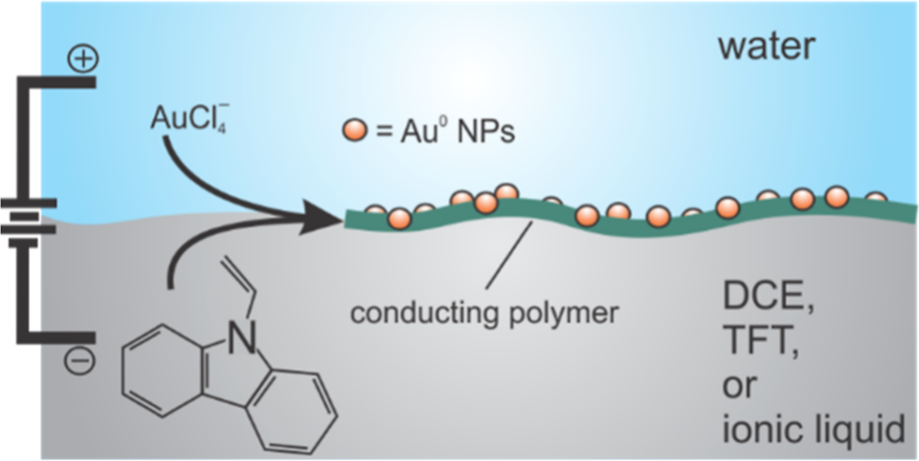

Metal nanoparticle (NP) incorporated conductive polymer films are attractive for their mechanical stability for biomedical applications and as heterogeneous electrocatalysis materials. Novel approaches to generate these materials with tunable properties are still being sought. Herein, the interface between two immiscible electrolyte solutions (ITIES) has been employed as a molecularly sharp and reproducible platform for simultaneous Au NP and poly(9-vinylcarbazole) generation. Three interfaces have been compared, including between water|1,2-dichloroethane (w|DCE), water|α,α,α-trifluorotoluene (w|TFT), and water|ionic liquid (w|IL). In this case the IL was P_8888_TB (tetraoctylphosphonium tetrakis(pentafluorophenyl)borate). 9-Vinylcarbazole (VC) can polymerize via two routes, either propagating through the vinyl substituent or the aryl rings. The former gives rise to a white semiconducting polymer with a wide bandgap, while the latter produces a green, conducting polymer. External potential control through voltammetric cycling was found to generate the film more rapidly favoring heterogeneous electron transfer with formation of the green poly(VC) variant at the ITIES. This was a free-standing film that could be easily removed from the interface. In the absence of external control, white polymer crystals formed within the oil phase spontaneously likely via AuCl_4_^–^ w → o transfer followed by a homogeneous electron transfer reaction mechanism. Scanning electrochemical microscopy probe approach curve experiments were used to quantify the electroactivity of the film and are complemented by direct conductivity measurements.

## Introduction

There is an increasing demand for novel polymer nanocomposites for applications such as personal electronics,^1−4^ biomedical/point-of-care devices,^5,6^ and energy storage/capture.^7^ Increasingly, there is a drive to create polymers with embedded inorganic, conducting, or dielectric nanomaterials to lend the polymer matrix mechanical support and/or reactive catalytic centers.^8,9^ Au NPs are often the focus owing to their high stability, biocompatibility, and ease of synthesis from HAuCl_4_ or KAuCl_4_ salts. The standard reduction potential for AuCl_4_^–^ is 1.002 V;^10^ thus, it has strong oxidizing behavior meaning it will readily react with most organic electron donors, which also means that Au^0^ is highly thermodynamically stable.

The interface between two immiscible electrolyte solutions (ITIES), e.g., between water|oil (w|o), has long been a platform for nanoparticle (NP) and nanocomposite synthesis.^11−14^ One of the seminal works was performed by Brust and Schiffrin;^11,12^ whereby, they demonstrated Au NP synthesis whose mechanism was later characterized by Uehara and Dryfe’s groups.^15−18^ Simultaneously, Johan’s et al.^19,20^ examined the thermodynamics and kinetics of NP formation at ITIES, while Cunnane’s group^13,21−23^ began earnest investigations of simultaneous metal NP and polymer film electrogeneration. More modern examples include the works of Nishi’s^24−28^ and Scanlon’s^29−32^ groups who have each developed 2D and 3D nanostructures at liquid|liquid interfaces often through external potentiometric control. In such an instance, the polymerization reaction can be controlled through the application of an external applied potential via an electrode immersed in either phase. This generates a potential drop, the Galvani potential difference (Δ_o_^w^ϕ = ϕ_w_ – ϕ_o_), localized across the ITIES.

Nishi et al.^28^ used a water|ionic liquid (w|IL) interface with AuCl_4_^–^(aq) and 2,2′:5′,2″-terthiophene (TT) in IL to generate Au NP/poly(TT) films with opposing structures growing on either side of the ITIES, i.e., a Janus-type film. The w|IL interface quickly became occluded with limited ion transfer; however, the growing film remained conductive and facilitated electron transfer from IL to w. Thus, Au NPs could continually be electrogenerated on the aqueous side forming flower-like shapes, while TT polymerization proceeded on the IL side generating polymer chains that extended into solution. Lehane et al.^32^ observed a similar phenomenon during electropolymerization of 3,4-ethyenedioxythiophene (EDOT), in which cerium sulfate, Ce(SO_4_)_2_(aq), acted as the electron acceptor. However, Ce NPs were not formed; instead, Ce^4+^ acts as a single electron donor (i.e., Ce^4+^ → Ce^3+^ + *e*^–^) and the PEDOT film was smooth on the aqueous side and rough on the oil side owing to continued polymer growth in that phase. Moreover, they have recently demonstrated that a potentiodynamic rather than a potentiostatic pulse program is necessary for effective film formation with voltammetric scan rates in the range of 25–100 mV s^–1^ required.^30^ These studies often employ a large ITIES (cm scale), while our group has explored the effects of miniaturizing the liquid|liquid interface to the micron scale (∼25 μm) toward NP and polymer film formation at both w|o^9,33−35^ and w|IL^36^ interfaces.

Our recent work at the micro-ITIES employed a modified pyrene molecule with two dithiafulvenyl (DTF) moieties tethered to either ends of the pyrene ring.^9^ By miniaturizing the interface, films as thin as ∼2 nm with Au nanoclusters (<1.7 nm) being formed. Large interfacial experiments resulted in rapid and uncontrolled Au NP/poly(bis(DTF)pyrene) formation. The majority of studies have used thiophene-based monomers such as EDOT^30,32^ or TT^23,28,33,34^ with a relatively straightforward polymerization mechanism. In the case of EDOT or TT, the α-carbon adjacent to the sulfur atom is the likely site for C–C bond formation.^37^ Moreover, aside from Nishi’s group,^24,25,27,28,38^ most employ molecular solvents rather than ILs, likely because ILs are expensive, difficult to purify, and technically challenging to work with owing to their high viscosity.

Herein, 9-vinylcarbazole (VC) polymerization has been investigated through an interfacial reaction at immiscible liquid|liquid interfaces between water|1,2-dichloroethane (w|DCE), water|α,α,α-trifluorotoluene (w|TFT), and w|IL. Tetraoctyphosphonium tetrakis(pentafluorophenyl)borate (P_8888_TB) IL is very hydrophobic and easily purified by exploiting its moderate melting point of ∼53 °C and recrystallizing it in ethanol or isopropanol at −20 °C.^39^ Both large (∼1 cm in diameter) and micro (25 μm diameter) interfaces were employed. VC is sufficiently hydrophobic as to remain in the oil or IL phase, while the gold salt, KAuCl_4_ was used as discussed above and acts as the oxidizing agent/electron acceptor in the aqueous solution. In this way, simultaneous Au NP generation and VC electropolymerization was performed.

**Scheme 1:**
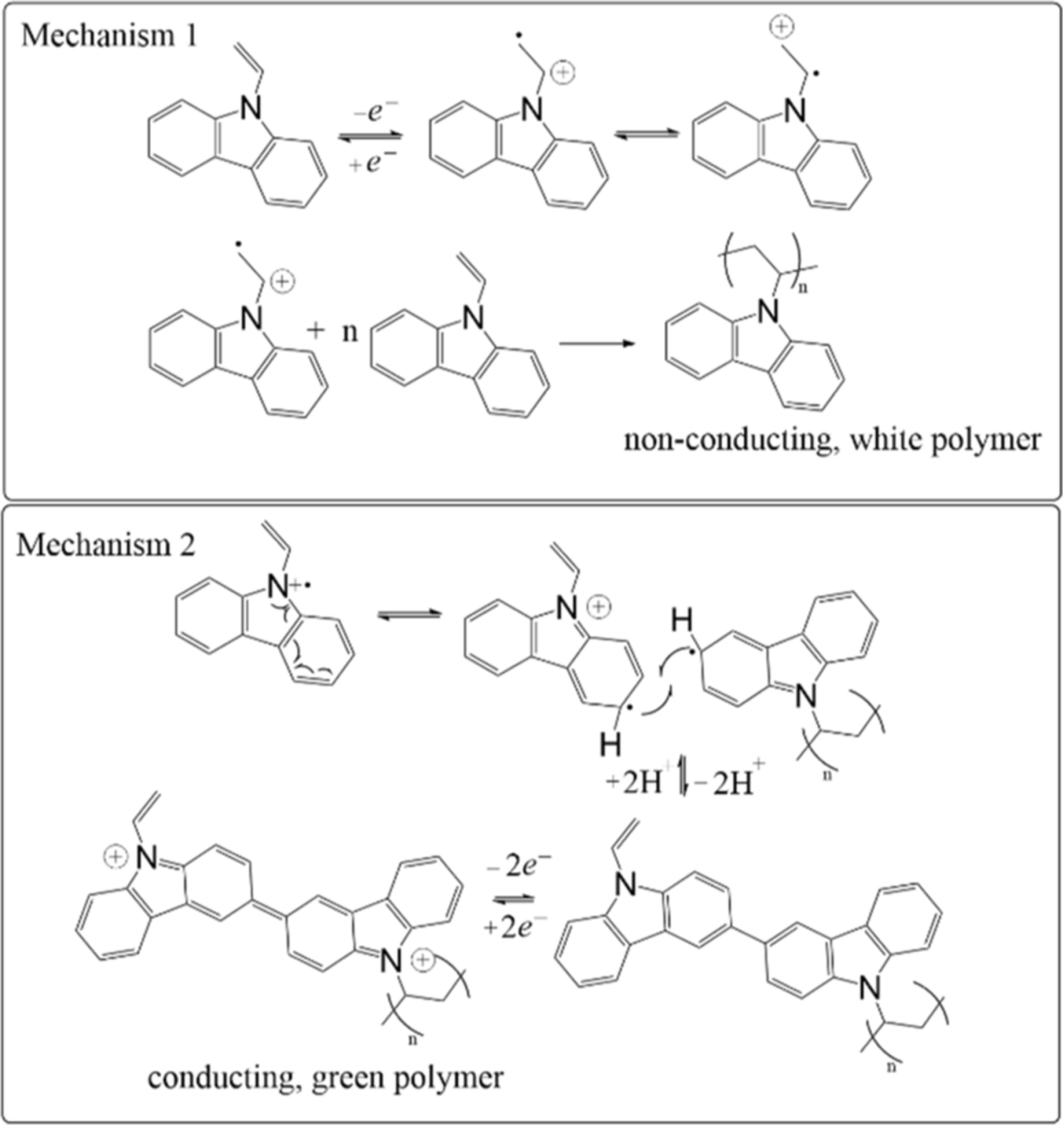
Chemical Structure and Polymerization Mechanism of VC Generating Either the Semiconducting, White (Mechanism 1) or Conducting, Green Polymer (Mechanism 2)

VC typically undergoes polymerization via the vinyl substituent (see mechanism 1, Scheme 1) which generates a white, semiconducting polymer.^40^ However, polymerization and cross-linking can also take place off the aryl rings as shown in mechanism 2 in Scheme 1; whereby, a green, conducting polymer is formed. The white one is a large bandgap semiconductor with modest hole transport properties^1,3^ which finds use as a composite layer in quantum-dot light emitting diodes (QD-LEDs) and organic solar cells (OSC).^3,7^ Carbazole containing conductive polymers have been used in capacitor^41^ and memory devices.^41,42^

At all 3 interfaces, green, conducting Au NP/poly(VC) was generated, however, only under external potential control. Importantly, a free-standing film was able to be removed from the ITIES for further analysis. Meanwhile, white, needle-like poly(VC) formed spontaneously with or without an applied potential and is likely the result of AuCl_4_^–^ spontaneous transfer from w → o (or IL), due to its intermediate hydrophobicity/hydrophilicity, followed by homogeneous reaction with VC in the oil/IL phase. This homogeneous reaction likely also acts as a driving force for AuCl_4_^–^ transfer. Scanning electrochemical microscopy (SECM) in the form of probe approach curve (PAC) experiments using an ultramicroelectrode (UME) were performed to investigate the electroactivity of films deposited on glass, insulating and conducting Au coated Si wafers. These data were compared to direct conductivity measurements. Water contact angle (WCA) measurements of films formed at the large ITIES between w|DCE and w|TFT were hydrophobic and showed no appreciable difference between the aqueous and organic sides of the as-prepared films.

## Experimental Section

All chemicals were used as received, unless otherwise indicated, with aqueous solutions prepared using Milli-Q ultrapure water (18.2 MΩ cm). 9-Vinylcarbazole (VC, 98%), potassium tetrachloroaurate (KAuCl_4_, >98%), 1,2-dichloroethane (DCE, ≥99.0%), α,α,α-trifluorotoluene (TFT, ≥99.0%), trioctylphosphine (>97%), and bromooctane (99%) were sourced from Sigma-Aldrich. Tetrakis(pentafluorophenyl)borate lithium etherate (LiTB, ≥99%) was purchased from Boulder Scientific. Potassium chloride (KCl, 99.0%) was obtained from ACP. TFT was distilled ∼3 times with activated charcoal to remove impurities, until anomalous peak current signals observed in blank w|TFT microinterfacial voltammetry disappeared. The IL P_8888_TB (tetraoctylphosphonium tetrakis(pentafluorophenyl)borate) was prepared as previously described.^39^

Microinterfacial experiments were conducted in the 2-electrode mode using the PG-618-USB potentiostat from Heka Electronics with the micropipette equipped inside a specialized holder described elsewhere.^9,33^ The working electrode (WE) lead of the potentiostat was coupled to an internal Au wire (Delta Scientific) via an SMA connector. The Au wire was immersed in the aqueous solution which was backfilled into the holder/capillary. A Pt wire (Heka Electroniks) was used as the counter/reference electrode (CE/RE) in the oil or IL phase. Unless otherwise indicated, the micro-ITIES was 25 μm in diameter.

Large-ITIES experiments employed a specialized 4-electrode cell^33,43^ and were measured using a CHI6059 potentiostat from CH Instruments. The Pt WE of the cell was electroplated with Au using a 5 mM KAuCl_4_(aq) solution until the wire changed color from silver to gold.

SECM experiments were conducted using the ElProscan (Heka Electroniks) in 3-electrode mode. The WE was a carbon fiber UME with a radius of *r* = 3.5 μm, while the CE and RE were Pt and Ag/AgCl wires, respectively. Probe approach curves (PACs) were performed at a tip speed of 1 μm s^–1^. UME fabrication details have been described recently.^44^

A JEOL JSM 7100 F equipped with energy dispersive X-ray (EDX) spectrometer was used for scanning electron microscopy (SEM) imaging; whereby, EDX spectra were analyzed via DTSA II software provided by the National Institute of Standards and Technology (NIST) in the US, see https://www.nist.gov/services-resources/software/nist-dtsaii.

The Tecnai Spirit transmission electron microscope (TEM) was employed for imaging Au NP/poly(VC) composite materials; whereby, samples were deposited on Au 200 mesh lacey carbon grids (Electron Microscopy Sciences).

Differential scanning calorimetry (DSC) was performed using the DSC-1 STAR* system (Mettler-Toledo), while thermogravimetric analysis (TGA) was conducted using a TA Instruments TGA55.

WCA measurements were made using an OCA 15EC contact angle instrument from Dataphysics.

## Results and Discussion

### Micro ITIES

**Figure 1:**
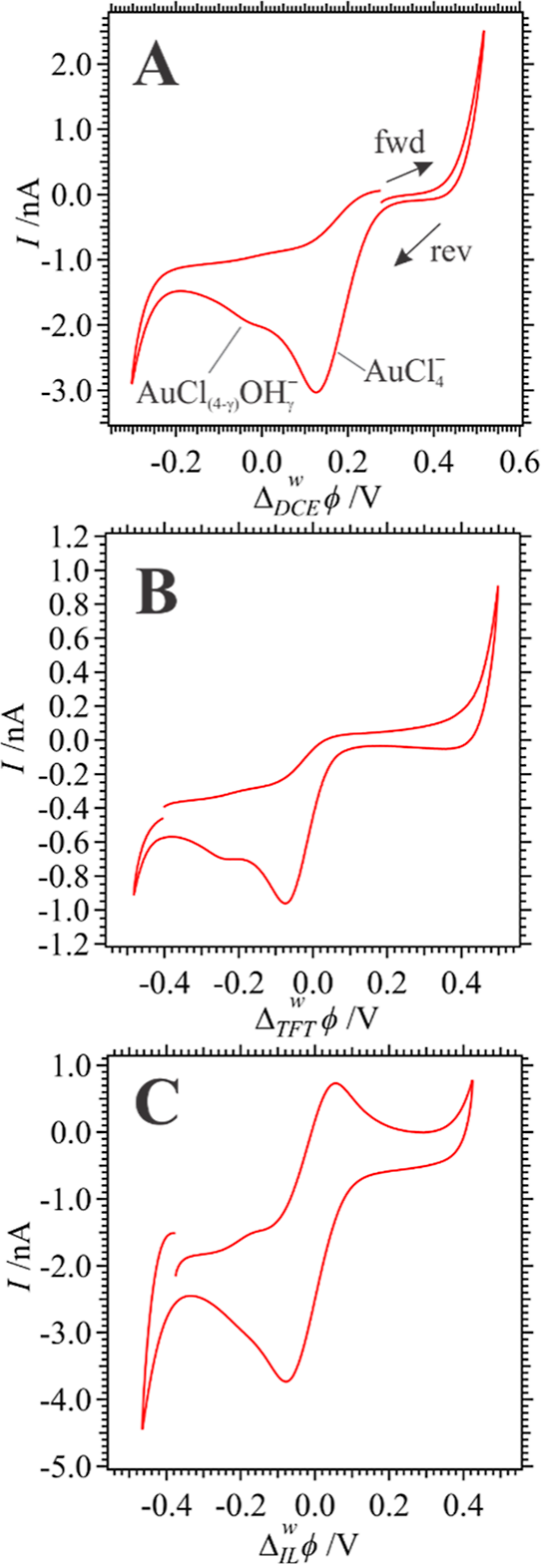
CVs recorded at a micro-ITIES with a scan rate (*v*) of 0.020 V s^–1^ using Cells 1 (A and B) or 3 (C) at water-DCE, TFT, and P_8888_TB interface in panels A, B, and C, respectively. See Scheme 2 for cell details. No VC was added to the oil or IL phase, i.e., these are blank CVs. Black arrows indicate scan direction for the forward (fwd) and reverse (rev) segments. Peaks in A have been labeled with the ion that is transferring from w → o.

**Scheme 2:**
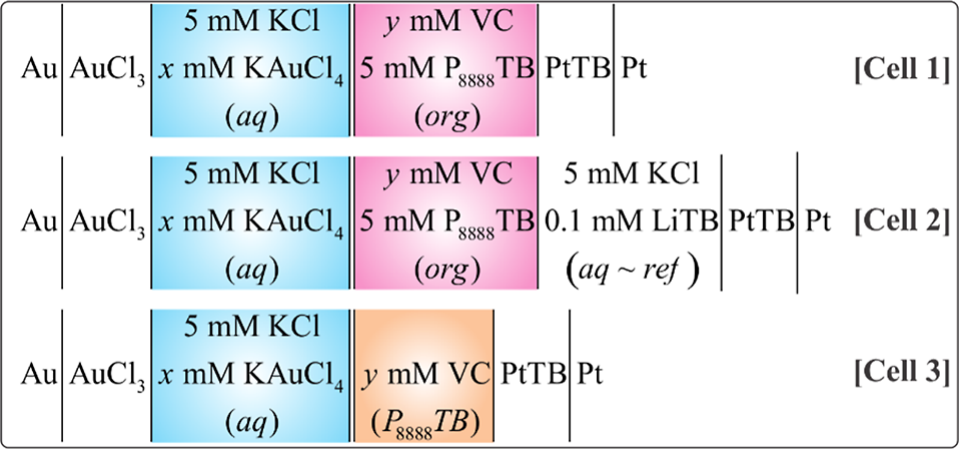
Electrolytic Cells Employed in Which *x* mM of KAuCl_4_(aq) and *y* mM of 9-Vinylcarbazole (VC) as Electron Donor and Monomer was Added the Organic Phase which was Either 1,2-Dichloroethane (DCE), α,α,α-Trifluorotoluene (TFT), or the Ionic Liquid (IL) Tetraoctylphosphonium Tetrakis(pentafluorophenyl)borate (P_8888_TB) Cells 1 and 3 represent the micro-interfacial experiments with the ITIES (25 μm in diameter) held at the tip of a specialized capillary, while Cell 2 was the large, 4-electrode ITIES cell (∼1 cm in diameter). The IL phase in Cell 3 was maintained at ∼60 °C using a water circulator (Polystat, Cole-Parmer) connected to a heating mantle. Double bars have been used to emphasize the polarizable liquid|liquid interface.

First, cyclic voltammograms (CVs) were obtained at a micro-ITIES (*r* = 12.5 μm) at either the w|DCE (Figure 1A), w|TFT (Figure 1B), or w|P_8888_TB (Figure 1C) interface with a scan rate of 0.020 V s^–1^ using Cells 1 or 3; see Scheme 2 for electrochemical cell descriptions. The w|IL interface in Cell 3 was maintained at ∼60 °C using a heating mantle attached to a water circulator, while all other interfaces were measured at room temperature (∼22 °C).

In Figure 1A, the limit of the polarizable potential window (PPW) was reached at roughly 0.5 and −0.3 V where a sharp increase and decrease in the current signal corresponding to the transfer of the supporting electrolyte ions K^+^ and Cl^–^, respectively, was observed. Two negative peak-shaped waves were recorded during the scan from positive to negative potentials with peak potentials (Δ_o_^w^ϕ_p_) of 0.126 and −0.009 V, which correspond to the simple ion transfer of AuCl_4_^–^ and AuCl_(4−γ)_OH_γ_^–^, respectively,^9,16,17,33,35^ from w → DCE. The presence of multiple Au anions agrees well with the ligand speciation of dissolved AuCl_4_^–^ with hydroxide at pH > 2 as has been previously shown;^35,45,46^ whereby, γ Cl^–^ ligands are replaced by OH^–^ ones. Assuming the following Nernstian, voltammetric curve relationship between the peak potential (Δ_o_^w^ϕ_p_) and the half-wave potential (Δ_o_^w^ϕ_1/2_) is true at the micropipette ITIES^47^
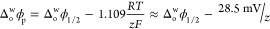
1in which *R*, *T*, and *F* have their usual thermodynamic significance, and *z* is the charge of the ion undergoing transfer. Then the respective Δ_DCE_^w^ϕ_1/2_’s for AuCl_4_^–^ and AuCl_(4−γ)_OH_γ_^–^ were calculated to be 0.154 and 0.020 V, using *z* = −1. The subscript “o” in eq 1 and above is used generically for the hydrophobic, oil phase meaning either DCE, TFT, or IL.

In the scan from negative to positive potentials, two sigmoidal waves were observed with Δ_DCE_^w^ϕ_1/2_ of −0.027 and 0.172 V (see Figure 1A). These represent the transfer of the Au anions back from DCE → w. The asymmetric CV profile observed is in good agreement with previous reports at a micro-ITIES held at the tip of a capillary;^35,48−50^ whereby, voltammetric asymmetry is caused by different diffusion regimes inside and outside the capillary.^51^ Ion transfer from inside to outside of the pipet (w → o, in this case) is under a linear diffusion regime generating peak-shaped responses, while ingress of ions from outside to inside the pipet (i.e., o → w transfer) results in sigmoidal waves due to the hemispherical diffusion regime similar to a reversible redox signal at an UME. Δ_DCE_^w^ϕ_1/2_ determined from the sigmoidal waves are somewhat shifted compared to the values obtained from the peak-potentials and eq 1 which may indicate the presence of a small uncompensated ohmic solution resistance (i.e., *iR*_s_ drop). This may be owing to stray capacitances within the microcapillary;^52^ however, the CV curves appear well shaped and the resistances were considered negligible. Nevertheless, these data are in good agreement with previous results obtained at a w|DCE interface by us^33,35^ as well as Uehara et al.^16,17^

Similarly, the simple ion transfer of AuCl_4_^–^ and AuCl_(4−γ)_OH_γ_^–^ were also recorded at a w|TFT micro-ITIES as shown by the CV in Figure 1B. The potential at the w|TFT interface was referenced using the formal ion transfer of tetramethylammonium (TMA^+^). Δ_TFT_^w^ϕ_1/2,TMA_ was determined to be 0.193 V (see Figure S1 of the Supporting Information) which is in good agreement with the formal TMA^+^ transfer potential reported by Olaya et al.^53^ TMA^+^ transfer was then used to determine the formal ion transfer potential of Cl^–^ (), which was found to be −0.630 V, and subsequently used to determine Δ_TFT_^w^ϕ_1/2_ for the gold chloride anions. For complete details see Section S1 of the Supporting Information. Thus, Δ_TFT_^w^ϕ_1/2_’s for AuCl_4_^–^ and AuCl_(4−γ)_OH_γ_^–^ transfer depicted in Figure 1B at a w|TFT interface were determined to be −0.045 and −0.217 V, respectively, using the peak current signals and eq 1.

Next, Cell 3 was employed in which the traditional organic phase was replaced with a hydrophobic IL, P_8888_TB, without VC added. CVs at the w|P_8888_TB interface were referenced to the simple ion transfer of Cl^–^ with  ≈ 0.58 V^36^ (data not shown). During both positive and negative scan directions of the CV in Figure 1C, two peak-shaped waves were observed. Owing to the high viscosity of the IL phase and, thus, much lower diffusion coefficients, the typical hemispherical diffusion regime observed during ingress is reduced to a linear one. This limits the depth of ion penetration when transferring from w → IL.^36^ Thus, linear diffusion is present inside and outside the capillary and the *i*–*V* recordings are symmetrically peak-shaped. Therefore, these two coupled signals are associated with AuCl_(4−γ)_OH_γ_^–^ and AuCl_4_^–^ transfer similar to the w|DCE and w|TFT experiments. Midpoint potentials [(Δ_IL_^w^ϕ_p,fwd_ + Δ_IL_^w^ϕ_p,rev_)/2] for the ion transfers were calculated using the peak potentials from the positive (Δ_IL_^w^ϕ_p,fwd_) and negative (Δ_IL_^w^ϕ_p,rev_) sweeps and found to be −0.175 and −0.012 V for AuCl_(4−γ)_OH_γ_^–^ and AuCl_4_^–^ transfer, respectively. Because the AuCl_(4−γ)_OH_γ_^–^ associated signal is merged significantly with the AuCl_4_^–^ one, its midpoint potential is approximative. As described by Nishi et al.^54^ the relationship between Δ_IL_^w^ϕ_1/2_ and Δ_IL_^w^ϕ^o′^ can be written as
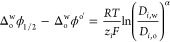
2Within which, *D*_*i*,w_ and *D*_*i*,o_ are the diffusion coefficients of ion *i* in the water or oil (DCE, TFT, or IL) phases, while α is a constant and equal to 0.5 for semi-infinite linear mass transfer or 1.0 for the spherical case. For the molecular solvents one can assume *D*_*i*,w_ ≈ *D*_*i*,o_; however, in the case of P_8888_TB this is not true with *D*_*i*,o_ often being several orders of magnitude smaller.^54^ Using Walden’s rule
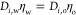
3where η_w_ and η_o_ are the viscosities of water (0.466 mPa s) and P_8888_TB (1206.2 mPa s)^55^ at 60 °C, one can approximate the diffusion coefficient ratio to be ∼2600. The viscosity of P_8888_TB was approximated from the literature value for trihexyltetradecylphosphonium tetrakis(pentafluorophenyl)borate, P_66614_TB, a structural isomer of P_8888_TB.^55^

Thus, using the Δ_IL_^w^ϕ_1/2_ values along with eq 2, Δ_IL_^w^ϕ^o′^ for AuCl_(4−γ)_OH_γ_^–^ and AuCl_4_^–^ were calculated to be −0.062 and 0.101 V for α = 0.5 or 0.213 and 0.051 V for α = 1.0, respectively. These values are in fair agreement with the values previously reported by us based on Δ_IL_^w^ϕ_1/2_ alone.^36^ The respective peak-to-peak separations were calculated to be 0.056 and 0.134 V for AuCl_(4−γ)_OH_γ_^–^ and AuCl_4_^–^. The latter is not unusual for voltammetric responses in an IL phase both at a liquid|liquid^36^ or a solid/solution^44^ interface in which peak-to-peak separations can be between 0.100 and 0.200 V. This is thought to be owing to the highly structured electric double layer (EDL) at solid/IL^56^ and w|IL^38^ interfaces; whereby, alternating ion-counterion layers can propagate several times into solution creating ionic multilayers. Cycling the potential forces a turnover in these multilayers which can have a very slow relaxation time inhibiting electron (or in this case, ion) transfer kinetics giving wider peak-to-peak separations than observed using conventional molecular solvents,^36^ i.e., ∼0.059 V.

**Figure 2:**
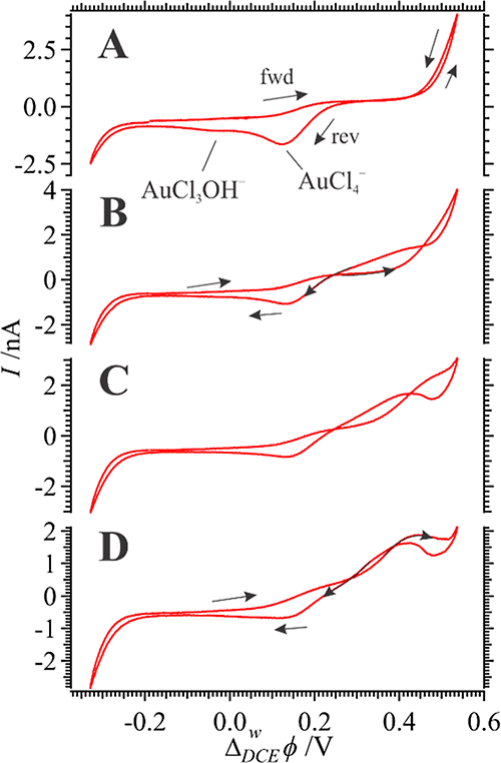
CVs recorded at 0.020 V s^–1^ using Cell 1 using a micro w|TFT interface with [VC]_TFT_ = 35 mM. Black arrows show the scan direction (“fwd” for the forward and “rev” for the reverse segments), and the scan number is indicated inset.

Subsequently, the effect of VC addition to each of the hydrophobic phases was investigated. Figure 2A depicts the initial CV recorded with [VC] = 35 mM in DCE using Cell 1 (Scheme 2). The CV was initiated at roughly −0.2 V and swept toward positive potentials with the sigmoidal AuCl_(4−γ)_OH_γ_^–^ and AuCl_4_^–^ transfer signals observed as described above. During the reverse scan, the *i*–*V* trace was observed to crossover at ∼0.4–0.5 V. Figure 2B–D show the subsequent 10th, 15th, and 25th scan. There was a concomitant positive current increase in the magnitude of the crossover current with increasing number of scans from 0.5 nA to almost 2 nA after 25 scans. Indeed, a peak current emerges during the positive sweep at ∼0.45 V by the 10th scan. Crossover events within *i*–*V* responses have been shown to be common during electrodeposition of electroactive material at micro and nano interfaces as demonstrated by us^34^ and Brasiliense et al.^57^ In the latter work,^57^ Co^2+^ was reduced onto a nanoelectrode generating a Co^0^ NP during the cathodic scan; however, upon reversal of the scan direction, Co^0^ was reoxidized. In our recent work,^34^ 2,2′:5′,2″-terthiophene (TT) was used as an electron donor and electropolymerized at a micro w|DCE interface with AuCl_4_^–^ acting as an electron acceptor. The AuCl_4_^–^ was simultaneously reduced generating Au NPs which were encapsulated within the growing polymer matrix.^34^ Thus, the crossover events were attributed to the dynamic and changing electroactive surface area of the growing liquid|solid|liquid junction. Alternatively, Heinze et al.^58^ referred to this as a “nucleation loop” and studied it at a large solid|solution interface; whereby, they attributed it to a comproprotionation reaction between the formed oligomers and as-yet unreacted monomer in the vicinity of the interface. In either case, these crossover events agree well with Au NP/poly(TT) electrogeneration at a miniaturized ITIES.^34^ As the CV scans increase there is a decrease in the current intensity for both AuCl_4_^–^ and AuCl_(4−γ)_OH_γ_^–^ ion transfer peaks indicating that either the Au salt is being consumed locally or that ion transfer is inhibited. Indeed, the AuCl_(4−γ)_OH_γ_^–^ transfer signal is not observable beyond the 15th scan. Similar voltammetric results were observed at the w|DCE micro-ITIES with [VC]_DCE_ = 5 and 25 mM (data not shown). Thus, it is proposed that the current signal observed at roughly 0.45 V is associated with an electron transfer event and the simultaneous electrogeneration of Au NPs and poly(VC).

**Figure 3:**
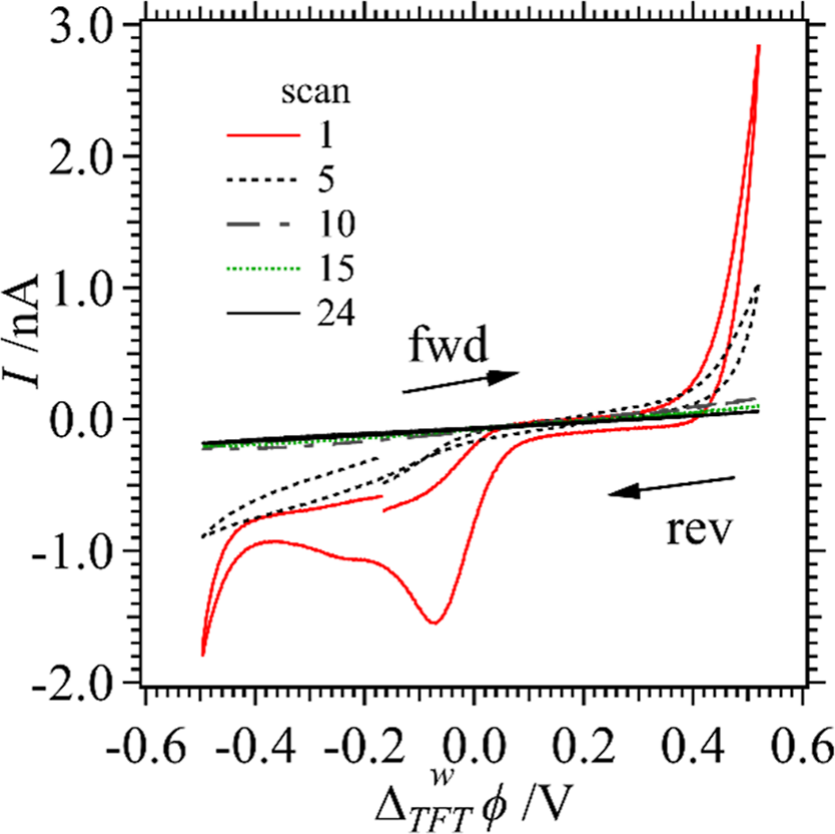
CVs recorded at 0.020 V s^–1^ using Cell 1 using a micro w|TFT interface with [VC]_TFT_ = 35 mM. Black arrows show the scan direction (“fwd” for the forward and “rev” for the reverse segments), and the scan number is indicated inset.

Next, the micro w|TFT interface was investigated with additions of VC to the TFT phase. Figure 3 shows the first and every subsequent fifth *i*–*V* scan recorded with [VC]_TFT_ = 35 mM. The first scan shows the distinct AuCl_4_^–^ and AuCl_(4−γ)_OH_γ_^–^ transfer signals; however, rapidly these ion transfer waves are suppressed and the entire voltammogram becomes resistive. Very similar CV results were observed at [VC]_TFT_ = 5 and 25 mM (data not shown). VC can undergo polymerization and/or cross-linking through two pathways as shown in Scheme 1.^40^ A white, nonconducting/semiconducting polymer is formed via vinyl polymerization; however, a green, conductive one forms with C–C bond formation off the aryl rings. Thus, owing to the increased resistance during voltammetric cycling at the micro w|TFT interface, it is likely that a nonconducting/semiconducting Au NP/poly(VC) composite forms or is otherwise blocked. Indeed, during prolonged experiments a white precipitate was observed in the TFT phase which were likely micro/nano-poly(VC) particles which formed due to spontaneous AuCl_4_^–^ transfer and homogeneous reaction in the oil phase.

All VC solutions were made fresh each day. If an organic solution (not in contact with the aqueous phase) was left to stand for more than a day, a white precipitate formed. This is hypothesized to be oxidation of the monomer in air generating poly(VC) micro/nanocrystals.

**Figure 4:**
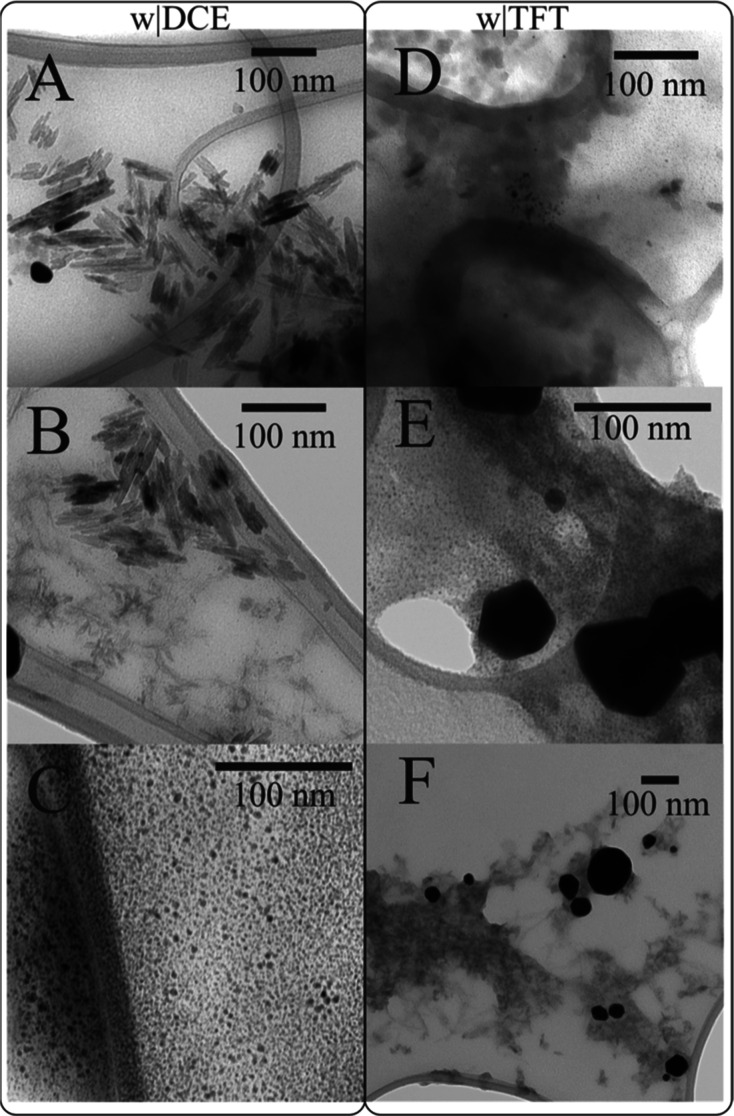
TEM micrographs taken of NP/polymer films deposited on a grid after 25 CV cycles were performed using Cell 1 with either DCE (A–C) or TFT (D–F) as the oil phase, as well as [VC]_org_ = 5 (top panels), 25 (middle panels), or 35 mM (bottom panels). A scan rate of 0.020 V s^–1^ was employed throughout.

Next, removing the pipet tip from the organic or IL solution, and using the syringe attached to the back of our modified pipet holder,^9^ a droplet of the aqueous phase was dispensed onto a TEM grid as well as a glass or silicon wafer for TEM/SEM and SECM imaging. Grids/wafers were then dried under a gentle flow of N_2_. Figure 4 shows the TEM micrographs obtained from a single droplet after 25 voltammetric scans using Cell 1 at either a w|DCE (Figure 4A–C) or w|TFT (Figure 4D–F). The initial VC concentration varied from 5 to 25 and 35 mM for the top to bottom panels, respectively.

When the organic phase was switched to TFT, comparatively smaller amounts of VC were needed to form the polymer film, versus the w|DCE interface, with no interfacial polymer nanocrystal formation observed at as low as [VC]_TFT_ = 5 mM. Figure S2 of the Supporting Information depicts the histograms developed by analyzing the TEM images using ImageJ software with a Gaussian fitting applied in which the peak-half-width-at-half-height was taken as one standard deviation (σ), with the peak position (*x*_0_) itself taken as the average NP size. Generally, NP diameters decreased concomitantly with increasing [VC] at both interfaces. At the micro w|DCE interface, as [VC] increased from 5 to 35 mM, the Au NPs decreased in size from 16 ± 20 nm to 2.5 ± 0.8 nm, while at the w|TFT interface, they decreased from 2.7 ± 0.3 nm to 1.6 ± 0.4 nm (see Figure S2). Increasing [VC] is likely associated with an increase in the kinetics of the electropolymerization reaction, meaning the polymer forms more quickly giving NPs less time to grow before they become encapsulated. Encapsulation in turn prohibits further NP growth. However, since the polymer film can still be conductive, electrons are free to transfer through the growing liquid|solid|liquid junction. Meaning, AuCl_4_^–^ and AuCl_(4−γ)_OH_γ_^–^ can continue to be reduced on the aqueous side, while VC is oxidized undergoing polymerization on the organic side.

**Figure 5:**
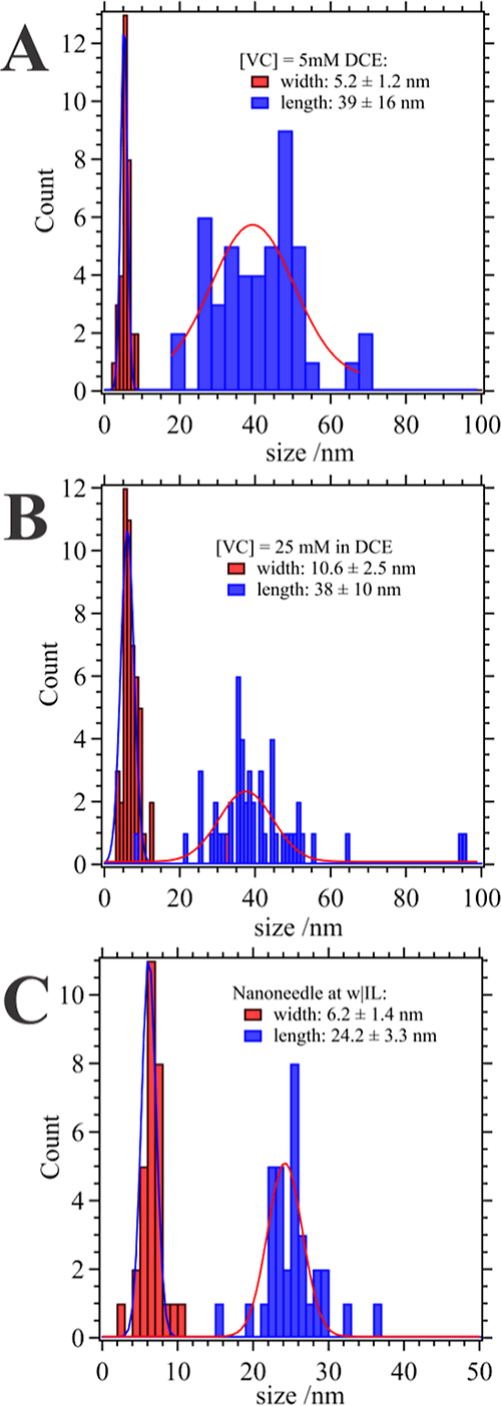
Histograms compiled from analyzing the TEM images of polymer nanoneedles depicted in Figure 4A,B, and figure B, measuring their width and length as indicated inset. Solid line traces are Gaussian fittings with peak listed as the mean size and half-width-at-half-height equal to σ.

Thus, at all VC concentrations at both w|o microinterfaces, larger NPs were observed as can be seen in Figure 4E,F which were often >100 nm. The nanoneedles present in Figure 4A,B are likely more crystalline and well ordered Au NP/poly(VC) formed at w|DCE interface due to the low rate of polymerization. These structures were not observed at higher concentrations of the monomer ([VC]_DCE_ = 35 mM in Figure 4C), only the relatively low dispersity spherical Au NPs were formed embedded within a continuous polymer matrix. The width and length of the nanoneedles were sized, and histograms plotted in Figure 5A,B. The width of the nanoneedles increased from 5.2 ± 1.2 nm to 10.6 ± 2.5 nm, while the length effectively remained the same at 39 ± 16 nm and 38 ± 10 nm for [VC] = 5 and 25 mM in DCE, respectively.

The larger Au NPs that have little or no poly(VC) associated with them are likely those formed on the aqueous side after the ITIES has been occluded by the polymer film. In this way, Au NP growth is uninhibited in the later stages of film generation. This Janus-type film growth agrees with the work of Nishi et al.^28^ as well as Lehane et al.^32^ described above. The presence of Au NPs versus the polymer matrix was confirmed using EDX spectroscopy (data not shown).

**Figure 6:**
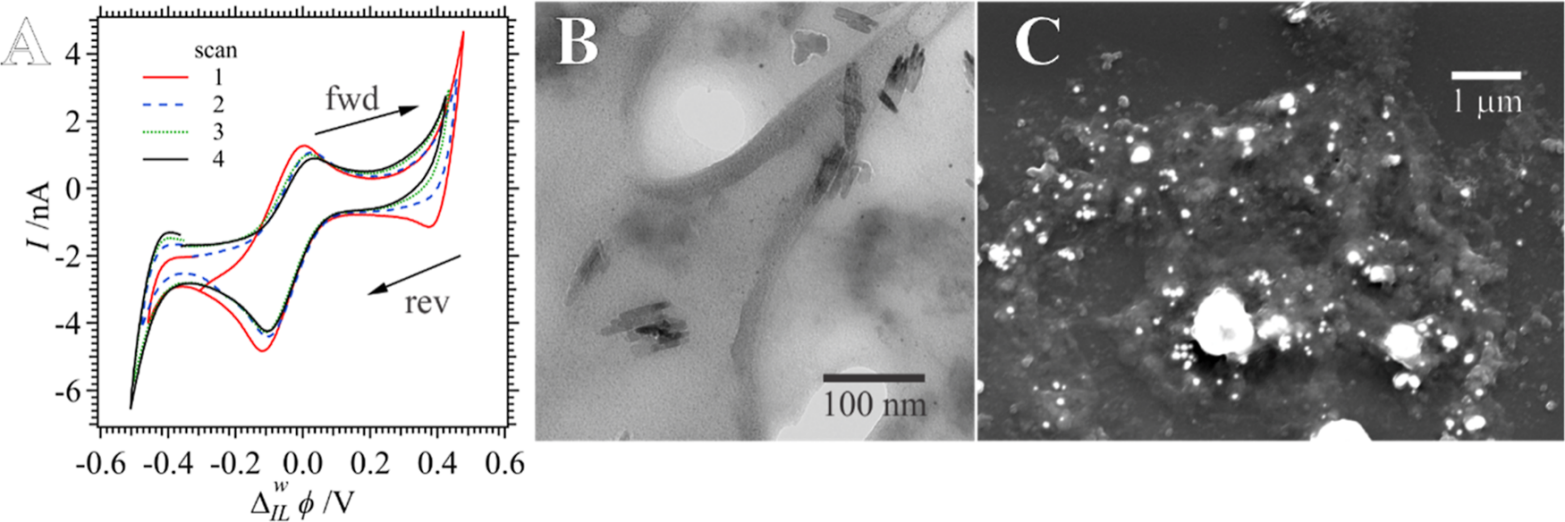
(A) Voltammograms recorded using Cell 3 (see Scheme 2) with 0.043 mol kg^–1^ of VC added to the P_8888_TB, IL phase performed at 0.020 V s^–1^ and 60 °C with the sweep number indicated inset. TEM (B) and SEM (C) micrographs of samples taken after the CV experiments in (A). A higher magnification TEM image from (B) has been provided in Figure S3 of the Supporting Information.

Subsequently, the micro w|IL interface was explored using Cell 3 (Scheme 2) at 60 °C in which 0.043 mol kg^–1^ (0.043 m) of VC was added to the P_8888_TB phase. Figure 6A shows the resultant *i*–*V* curves for the first 4 voltammetric scans. After 4 scans the w|IL interface became unstable and ruptured, either forming a droplet in the IL phase or retracting back up into the micropipette. This may be owing to a drastic change in the w|IL surface tension through the formation of the polymer and metal NPs at the ITIES. Similar instabilities were not observed at the w|DCE or w|TFT interfaces. The solubility of VC in P_8888_TB was poor; thus, higher concentration experiments were not possible and lower [VC] showed no appreciable change versus the blank CV shown in Figure 1C (data not shown). The low solubility of neutral compounds is not uncommon with some ILs which have been shown to preferentially dissolve ionic species; particularly, if they have an ion in common with the IL solvent phase.^59,60^ Thus, a focus of future work will be to functionalize an ionic liquid with an electropolymerizable moiety and pair it with a common ion of the bulk IL; however, this is beyond the present scope. After VC addition, the AuCl_(4−γ)_OH_γ_^–^ transfer signal has disappeared and after each scan there is a modest decrease in the symmetric peak-shaped response with Δ_IL_^w^ϕ_1/2_ = −0.058 V. While these data are somewhat ambiguous, they agree well with our recent results employing an IL containing a modified side chain on the phosphine core incorporating a ferrocene moiety;^36^ this was referred to as a ferrocene-ionic liquid (FcIL). In those experiments, FcIL was added to the P_8888_TB phase and underwent heterogeneous electron transfer with AuCl_4_^–^, reducing it to form Au NPs. As the FcIL concentration increased, the voltammetric peak current of the electron transfer wave increased and the AuCl_(4−γ)_OH_γ_^–^ transfer wave disappeared. Thus, these data presented in Figure 6A agree well with these previous results.^36^

After the *i*–*V* experiments demonstrated in Figure 6A, the micropipette was removed from the IL solution and, similar to the water|molecular organic solvent experiments described above, a drop was ejected from the pipet onto a glass slide and TEM grid. Figure 6B,C show the TEM and SEM images recorded, respectively. From the TEM image, both small and needle-like nanoparticles can be observed. Figure 5C shows histograms of the size distribution, both length and width, of the nanoneedles formed. These were comparable to those generated at the w|DCE and w|TFT interfaces. Au NPs were assumed to be the dark spherical particles in the TEM images, and a histogram of their size distribution has been plotted in Figure S2G of the Supporting Information. Interestingly, Au NPs formed at w|IL were much smaller than those electrogenerated at the other two interfaces at a size of 0.8 ± 0.4 nm. The supramolecular environment of ILs tends to favor the growth of small, low dispersity NPs.

### Large ITIES

**Figure 7:**
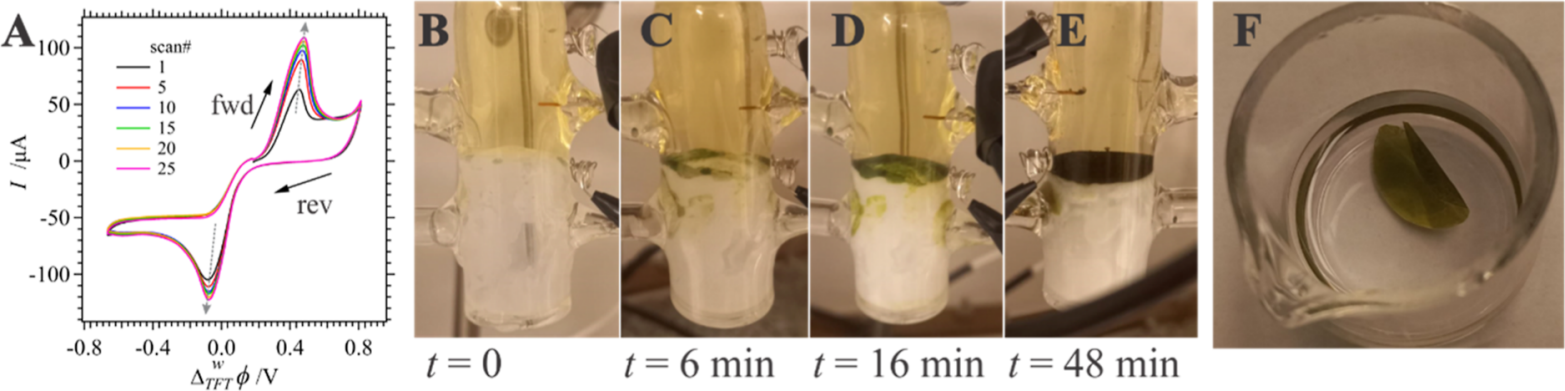
(A) CVs recorded at a large ITIES using Cell 2 (see Scheme 2) with TFT as the organic phase with 5 mM KAuCl_4_(aq) and 20 mM VC(TFT). Black arrows indicate scan direction, while gray, dashed ones track the evolution of the peak intensity with scan number. (B–E) Photographs taken of the large ITIES during the voltammetric scans shown in (A) at the times indicated. (F) Image taken of the final Au NP/poly(VC) nanocomposite suspended in isopropanol.

Figure 7 shows the results obtained at a large interface using TFT as an example; similar data were obtained at a w|DCE one. CVs have been plotted in Figure 7A such that an hypothesized electron transfer wave appears at an Δ_TFT_^w^ϕ_ET,1/2_ ≈ 0.45 V and an irreversible AuCl_4_^–^ transfer peak occurs with  = 0.045 V. As discussed above, the irreversible AuCl_4_^–^ transfer signal is likely indicative of the Au salt reacting homogeneously with VC generating the Au NP/poly(VC) nanocrystals in the TFT phase. The rapid presence of a white precipitate that gradually accumulates in the TFT phase (see Figure 7B–E) agrees well with this hypothesis. With the application of an external electric field, however, a green film gradually grows to occlude the w|TFT interface as can be seen in photos displayed in Figure 7B–E. After 25 cycles at 0.020 V s^–1^, a green film was lifted from the surface and deposited into a vial containing isopropanol (Figure 7F). If the film is not kept suspended in an organic solution, it tends to curl up into itself.

A large w|IL interface was not attempted as they have been shown to be highly resistive;^28^ however, an aspect of future work will be to design a specialized large-ITIES cell to mitigate these effects similar to one prepared by Cunnane’s group.^23^

**Figure 8:**
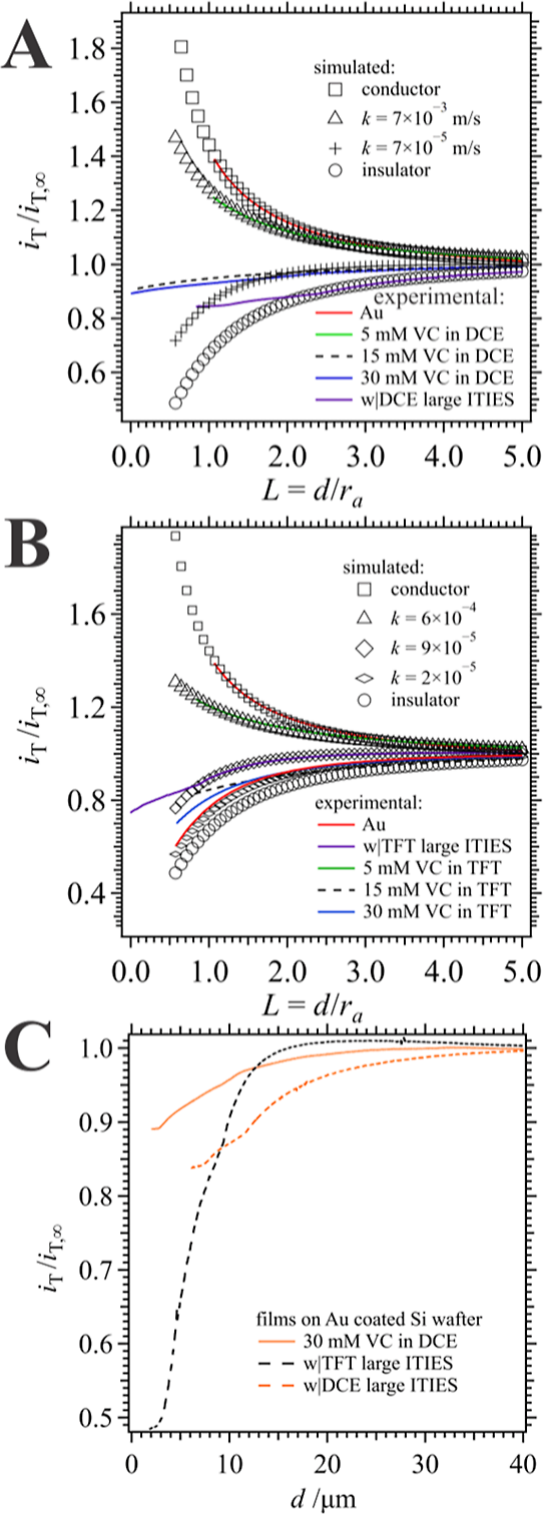
Experimental (line traces) SECM PACs toward films electrogenerated at w|DCE (A) and w|TFT (B) interfaces at different [VC], as indicated inset, and deposited on a Au coated silicon wafer. All films were generated using CV with 25 scans at 0.020 V s^–1^ at the micro-ITIES unless indicated otherwise. The currents have been normalized, i.e., tip current (*i*_T_) divided by the tip current at >100 μm tip-to-substrate distance (*i*_T,∞_). Simulated PACs (marker traces) generated using a custom finite element simulation (Comsol Multiphysics, version 6.2) as indicated inset. (C) PACs from (A and B) plotted against *d* to emphasize inflection points along the current trace where it is suspected that the UME makes contact with the Au NP/poly(VC) film.

PACs were then obtained using a 0.9 mM ferrocene methanol (FcCH_2_OH) in 5 mM KCl aqueous solution toward Au NP/poly(VC) films deposited on a Au coated Si wafer or glass substrate and electrogenerated at the w|DCE or w|TFT interfaces are shown in Figure 8A,B, respectively; whereby, the tip potential was maintained at ∼0.6 V, sufficient to oxidize FcCH_2_OH. PACs were compared to ones simulated using Comsol Multiphysics; for simulation details see the Supporting Information Section S4. The current recorded at the UME tip (*i*_T_) has been normalized with respect to the tip current far from the substrate (*i*_T,∞_), while the *x*-axis, tip-to-substrate distance (*d*) has been normalized via the UME radius (*r*_a_ = 3.5 μm), i.e., *L* = *d*/*r*_a_. Additionally, the *R*_g_ or ratio of outer glass radius (*r*_g_) to *r*_a_ (*R*_g_ = *r*_g_/*r*_a_) was determined be ∼3 through comparison of the experimental PAC (red trace in Figure 8A) toward a bare Au/Si wafer to a simulated one (□ curve in Figure 8A), which showed good overlap.

Intermediate feedback conditions were simulated using a flux condition at the substrate boundary with *k* as the redox mediator heterogeneous, rereduction rate constant. The green trace in Figure 8A shows the response for a film electrogenerated using 5 mM of VC in DCE, which matches well with a simulated PAC using *k* = 7 × 10^–3^ m s^–1^ (see Table 1). However, with increasing [VC] the insulating behavior of the film deposit increases and inflection points within the experimental PACs were observed. A similar trend was observed for films created at the w|TFT interface.

**Table 1:** Surface Rate Constant (*k*) Determined from Overlaying Simulated PACs Over Experimental Ones Shown in Figure 8

[VC]org/mM	*k*/m s^–1^ (w|DCE)	*k*/m s^–1^ (w|TFT)
5	7 × 10^–3^	6 × 10^–4^
15	7 × 10^–5^	2 × 10^–5^
30	7 × 10^–5^	3 × 10^–5^
large ITIES (aq side)	insulating	2 × 10^–5^

To emphasize the presence of these infection points, PACs with noticeable changes in slope have been plotted in Figure 8C without normalizing the *x*-axis/tip-to-substrate distance. We hypothesize that these changes in the slope of the PAC are due to the tip coming in contact with the film and penetrating or compressing it. Since the film is moderately conducting as well as soft and porous, as the UME makes direct contact (possibly entering inside of it) the tip would experience a new diffusion regime, or alternatively, oxidize the polymer directly. Nevertheless, by performing a PAC toward the bare conducting substrate adjacent to the film deposit and comparing the difference in *d* values one can obtain a semiquantitative measurement of the film thickness. The CCD camera attached to the magnifying lens assembly of the ElProscan was used to confirm the tip position over the film deposit. Only films formed at the w|DCE micro-ITIES at 15 and 30 mM of VC, as well as at both large interfaces demonstrated PACs with these kinds of responses. In all cases, film thicknesses were roughly 60–70 μm. 5 mM VC in DCE and all VC concentrations at the w|TFT micro interface showed no appreciable changes in slope, and it was not possible to characterize them in this way.

Comparing the PAC measurements from films formed at the micro w|DCE and w|TFT interfaces, the former had the lowest electroactivity (see Table 1) when [VC] was 15 or 30 mM. For the 5 mM [VC] case, the micro w|DCE interface was evaluated at *k* = 7 × 10^–3^ m s^–1^, while the w|TFT one was found to have *k* = 6 × 10^–4^ m s^–1^. While all samples showed good surface coverage, the porous nature of the film may only slightly impede access of the redox mediator to the Au coated surface. Thus, the thin-film effectively mimics the Au coated surface kinetically more closely.

Even for Au NP/poly(VC) composites formed at large ITIES, the w|TFT film demonstrated modest electroactivity with *k* = 2 × 10^–5^ m s^–1^, while at w|DCE the material was almost entirely insulating. However, both large ITIES films were green and showed good surface coverage upon visual inspection. Large ITIES generated films deposited on a conducting Au slide showed similar PAC behavior whether the film was positioned with the aqueous or organic phase side up. The data shown in Figure 8 is for aqueous side. Similarly, electrogenerated Au NP/poly(VC) composites applied to an insulating, glass substrate demonstrated near perfect insulating behavior; however, large w|DCE films exhibited modest conducting behavior (see Figure S5 of the Supporting Information). The w|DCE films also showed the slight change in slope which, as described above, may indicate the UME contacting the polymer film. There was a slight increase in electroactivity for the aqueous side versus the organic side of the w|DCE film which may be owing to presence of the larger Au NPs.

**Figure 9:**
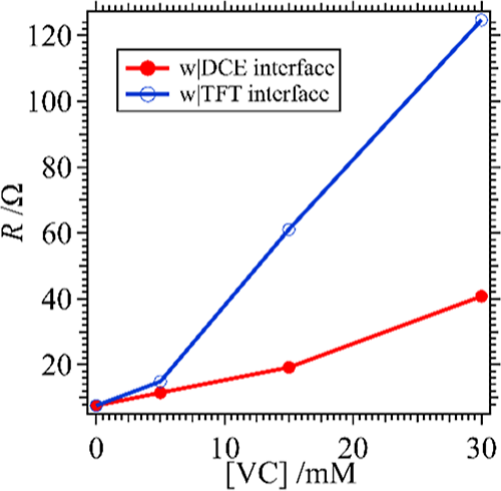
Plot of film resistance versus [VC] for Au NP/poly(VC) films generated at a micro w|DCE (red, solid circles trace) or w|TFT (blue, open circles curve) interface as described in Figures 2 and 3.

The conductivity of the films generated at micro-ITIES’s was measured directly in air via voltammetry by depositing them onto a 4 mm diameter glassy carbon electrode (GCE) while contacting a 25 μm diameter inlaid disc Pt UME, as described recently,^9^ employing them as CE/RE and WE, respectively. Figure 9 is a plot of the measured resistance versus the amount of VC used in either the DCE or TFT phase. Overall, the film resistances (*R*_film_) were low, below ∼125 Ω, with those generated at the DCE interface showing the lowest resistances. Assuming a film thickness of ∼60 μm, then the conductivity (*C* = 1/*R*_film_) decreases from 2200 to 130 S m^–1^ for w|TFT films, while it transitions from 2200 to 410 S m^–1^ for w|DCE ones.

Thus, these data suggest that Au NP/poly(VC) films are more conductive when electrogenerated at a micro w|DCE interface compared to a w|TFT one. The trend of increasing *R*_film_ with increasing [VC] aligns with the trend in decreasing electroactivity observed during PAC measurements. We hypothesize that the incorporation of polymer nanoneedles may play a role in enhancing the overall film conductivity. Nuraje et al.^61^ were able to prepare Fe NP coated nanoneedles with fast conductance switching at a water|chloroform interface using polyaniline and polypyrrole. To form the highly crystalline nanoneedles, they had to greatly reduce the FeCl_3_ oxidant and monomer concentrations so that the rate of the polymerization was in turn slowed. The results here further demonstrate the vital role of solvation on polymerization and that it can be transposed to electrochemically controlled methodologies.

Intermolecular interactions between the solvent and growing polymer contribute to its solubility, including some of the most common dipole–dipole, hydrogen bonding, and dispersion forces.^62−64^ It is possible that the poor solubility of poly(VC) in TFT may be owing to π–π interactions that help stabilize the growing polymer, aiding in its growth and rapid precipitation. Hansen solubility parameters are one route to quantitatively understand these interactions; however, this approach is beyond the scope of the present work.

**Figure 10:**
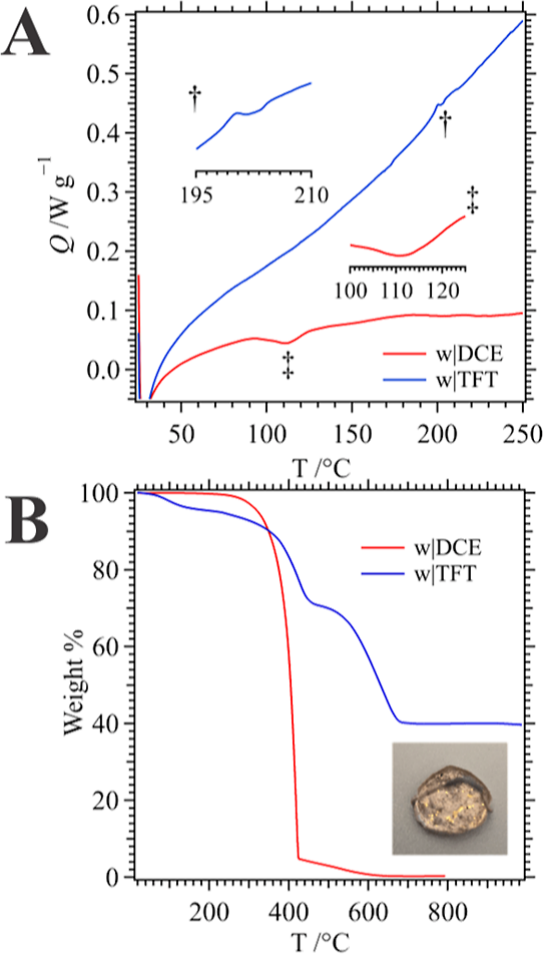
(A) Differential and (B) TGA curves of Au NP/poly(VC) nanocomposites electrogenerated using ∼20 voltammetric scans at 0.020 V s^–1^ at a large ITIES, Cell 2 (see Scheme 2) between w|TFT (―, blue trace) or w|DCE (―, red trace) acquired at 5 °C/min. Inset in (A) are regions of each curve highlighting the likely glass transition (*T*_g_) of each nanocomposite, while in (B) a photograph taken of the TGA dish post analysis shows a large amount of gold particles. In DSC experiments, samples were initially heated to 100 °C then cooled to room temperature to remove and “thermal history” from the material.

Figure 10A,B depict differential thermograms and TGA curves recorded using Au NP/poly(VC) composites electrogenerated at a large ITIES between either w|DCE or w|TFT using Cell 2 with [VC]_org_ = 35 mM and ∼20 voltammetric scans at 0.020 V s^–1^. In DSC experiments, the samples were first quickly heated to 100 °C and then cooled to room temperature to remove any thermal history from the nanocomposite. The w|DCE film DSC experiments showed an endothermic transition at roughly 105 °C, while the w|TFT one had a minor transition at 201 °C which we propose to be the glass transition temperatures (*T*_g_). These results are very different than those reported by Xu et al.^65^ for a diphenylphosphine oxide modified poly(VC). However, the difference may be linked to the level of crystallinity in each sample. The w|DCE micro-ITIES results described above suggest that highly crystalline nanoneedle formation is favored while the opposite was the case for the w|TFT interface. Moreover, higher *T*_g_ for the w|TFT generated composite is indicative of a higher degree of cross-linking; whereby, polymer mobility is restricted and only observed at higher temperatures, thus requiring more heat to transition.

TGA analysis shows a large mass loss starting at roughly 240 °C for the large w|DCE ITIES electrogenerated films indicating the general thermal decomposition of the polymer nanocomposite (Figure 10B). Based on the residual plateau, ∼5 wt % of the w|DCE film is composed of Au. Meanwhile, the film created at the large w|TFT interface had 3 thermal degradation periods at 58–150, 260–470, and 500–710 °C. The first is likely owing to moisture loss, while the second is from the degradation of the phenyl ring,^66^ while the final transition is the complete loss of the polymer network. The absence of this second phenyl ring degradation from the w|DCE film seems to agree with the lower *T*_g_ that indicated less cross-linking and thus less phenyl ring activation during electropolymerization. Interestingly, the residual plateau indicates a high 39 wt % Au content within the nanocomposite. Inset in Figure 10B is a photo taken of the TGA crucible after decomposition of the w|TFT nanocomposite. One can observe the high amount of metallic Au present in the dish. Despite the higher Au content, the w|TFT nanocomposite showed lower conductivity. Thus, smaller, isolated Au NPs likely do not contribute very much to the overall conductivity of the material.

Finally, the hydrophobicity of films electrogenerated at large w|DCE and w|TFT were of sufficient size to examine using WCA measurements. Figure S6 (see the Supporting Information) depicts the images acquired after a 2 μL drop of ultrapure water was dispensed on either the aqueous or organic phase side of the film. The w|TFT films demonstrated slightly higher WCAs of ∼106° for the aqueous side and 108° for the organic one, while w|DCE films showed an average of 99.1° for the water side and 101.5° for the side formed toward the oil phase. Once removed from the isopropanol solution, both films became brittle, showing poor mechanical stability. The higher effective hydrophobicity of the w|TFT film is interesting since it showed consistently higher Au content from TGA analysis. This may mean that the Au NPs are well integrated within the polymer matrix.

## Conclusions

Three immiscible liquid|liquid interfaces, including w|DCE, w|TFT, and w|P_8888_TB, have been compared toward simultaneous electrosynthesis of Au NP/poly(VC) film formation. The cross-linked green, conducting polymer, was only observed during interfacial, heterogeneous electron transfer reactions with modest conductivity and electroactivity. The latter was determined through SECM PAC experiments with comparison to simulated PACs generated in a specialized finite element analysis code in the Comsol Multiphysics software environment. Au NP/poly(VC) films generated at w|DCE and w|TFT interfaces mimicked either the conductive or insulating substrate they were deposited on. As the coating thickness increased, the vertical conductivity through the film decreased along with its electroactivity when deposited on a conductive, Au coated Si wafer substrate.

The w|TFT interface was found to more easily generate the cross-linked nanocomposite at modest monomer concentrations, e.g., ∼5 mM, which was a less conductive nanocomposite than the highly crystalline nanoneedle incorporated films generated at a w|DCE one. Thus, it is likely that TFT is a better solvent toward poly(VC) oligomers facilitating the formation of smaller, low dispersity NPs and that the kinetics of this reaction are much higher versus those at a w|DCE interface. Exploiting the solvent effect, one can more easily generate metal NP embedded conductive polymer films and tailor them to a particular application. DSC thermograms showed small endothermic *T*_g_ phase transitions at 105 and 201 °C for films electrogenerated at the respective w|DCE and w|TFT interfaces. The higher *T*_g_ likely indicates greater cross-linking for the w|TFT film.

The poor solubility of VC in the IL phase limited the depth of study at the w|IL interface; however, the supramolecular IL structure likely contributes to the comparatively small Au NPs this interface was able to electrogenerate. This agrees with our previous work electrosynthesizing Au NPs using ferrocene (Fc) as well as FcILs at a micro w|IL interface.^35,36^ To investigate wider monomer concentration regimes, it is necessary to develop an electropolymerizable IL to overcome this solubility bottleneck.

This work highlights the need to carefully select the organic or IL solvent for this type of interfacial electrosynthetic method development and take into consideration monomer and polymer/nanocomposite product solvent interactions.

## Supporting Information Available

Voltammetric details of AuCl_4_^–^ simple ion transfer at a w|TFT interface; NP sizing results; SECM Comsol simulation description; and WCA measurements (PDF)

## Abbreviations

1,2-dichloroethane: (DCE)
2,2′:5′,2″-terthiophene: (TT)
3,4-ethyldioxythiophene: (EDOT)
9-vinylcarbazole: (VC)
α,α,α-trifluorotoluene: (TFT)
cyclic voltammogram: (CV)
dithiafulvenyl: (DTF)
electric double layer: (EDL)
interface between two immiscible electrolyte solutions: (ITIES)
ionic liquid: (IL)
nanoparticle: (NP)
organic solar cells: (OSC)
poly(3,4-ethyldioxythiophene): (PEDOT)
probe approach curve: (PAC)
quantum-dot light emitting diodes: (QD-LEDs)
scanning electrochemical microscopy: (SECM)
tetrakis(pentafluorophenyl)borate lithium etherate: (LiTB)
tetraoctylphosphonium: (P8888+)
tetraoctylphosphonium tetrakis(pentafluorophenyl)borate: (P8888TB)
ultramicroelectrode: (UME)
water|ionic liquid: (w|IL)
water|1,2-dichloroethane: (w|DCE)
w|α,α,α-trifluorotoluene: (w|TFT)
