# Supplementary material for: Comparison of Au Nanoparticle/Poly(9-vinylcarbazole) Thin-Film Electrogeneration at 3 Distinct Liquid/Liquid Interfaces: Water/1,2-Dichloroethane, /α,α,α-Trifluorotoluene, Or/Ionic Liquid
Source: Langmuir. 2024 Nov 5;40(46):24494–506. doi: 10.1021/acs.langmuir.4c03265 (PMC11580744; doi:10.1021/acs.langmuir.4c03265)
Supplement: Supplementary file 1 — la4c03265_si_001.pdf [file la4c03265_si_001.pdf]

Supplementary Information for: Comparison of Au nanoparticle/poly(9-vinylcarbazole) thin-film electrogeneration at 3 distinct liquid/liquid interfaces: water/1,2-dichloroethane, /*α,α,α*-trifluorotoluene, or /ionic liquid

Leila Nazari and Talia Jane Stockmann*

Department of Chemistry, Core Science Facility, Memorial University of Newfoundland, 45 Artic Ave, St. John's, NL, Canada, A1C 5S7

Table of Contents

Table of Contents 1
1.0 Formal ion transfer potential of $AuCl_4^-$ determined at a w|TFT micro interface 2
2.0 Nanoparticle sizing results 3
3.0 Comsol simulation details and scanning electrochemical microscopy (SECM) results 4
4.0 Water contact angle measurements of as-prepared films at large-ITIES 6
5.0 References 7

1.0 Formal ion transfer potential of $AuCl_4^-$ determined at a w|TFT micro interface

To determine the formal ion transfer potential of $AuCl_4^-$ and $AuCl_3OH^-$ the following electrolytic cell was employed in which ~1 mM of tetramethylammonium chloride (TMACl), $z \approx 1$, was added to the aqueous phase.

$$\mathrm{Au}\left|\mathrm{AuCl_3}\right|\begin{array}{c}\text{5 mM KCl}\\ z\text{ mM TMACl}\\ (aq)\end{array}\left\|\begin{array}{c}\text{5 mM }\mathrm{P_{8888}TB}\\ (TFT)\end{array}\right|\mathrm{PtTB}\left|\mathrm{Pt}\right. \quad \text{[Cell S1]}$$

Scheme S1: Additional micro-ITIES electrochemical cell used to perform reference voltammograms.

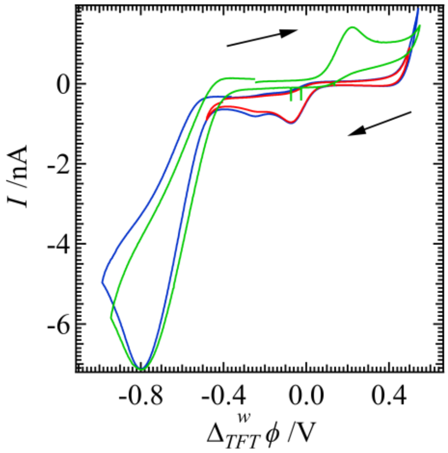

Figure S1: CVs recorded using Cell 2 (red and blue traces) with no 9-vinylcarbazole (VC) added to the TFT phase (see the main text) and Cell S1 (green trace, see Scheme S1 for cell details) with ~1 mM of tetramethylammonium chloride (TMACl) in the aqueous phase. A scan rate of 0.020 V s^{-1} was used.

–

Figure S1 depicts the *i*-*V* responses recorded using Cells 2 and S1 at the w|TFT micro interface with no VC added to the TFT phase. The TMA^+ transfer wave can be seen with a positive peak signal at 0.2215 V indicating a half-wave potential of $\Delta_{TFT}^{w}\phi_{1/2,\mathrm{TMA}^+}$ = 0.193 V; this is in good agreement with the value reported by Olaya *et al.*[1] The negative peak potential for Cl^- transfer from *w*→*TFT* was observed at –0.788 V; meanwhile, the half-wave potential $\left(\Delta_{TFT}^{w}\phi_{\mathrm{Cl}^-,1/2}\right)$ obtained from the sigmoidal return wave for the green and blue curves was determined to be roughly –0.630 V. The value measured from the sigmoidal wave was assigned as the formal Cl^- transfer potential, $\Delta_{TFT}^{w}\phi_{\mathrm{Cl}^-}^{o'}$, and used to calculate the formal ion transfer potential of $AuCl_4^-$ and $AuCl_3OH^-$ at the w|TFT interface to be –0.045 and –0.217 V, respectively.

2.0 Nanoparticle sizing results

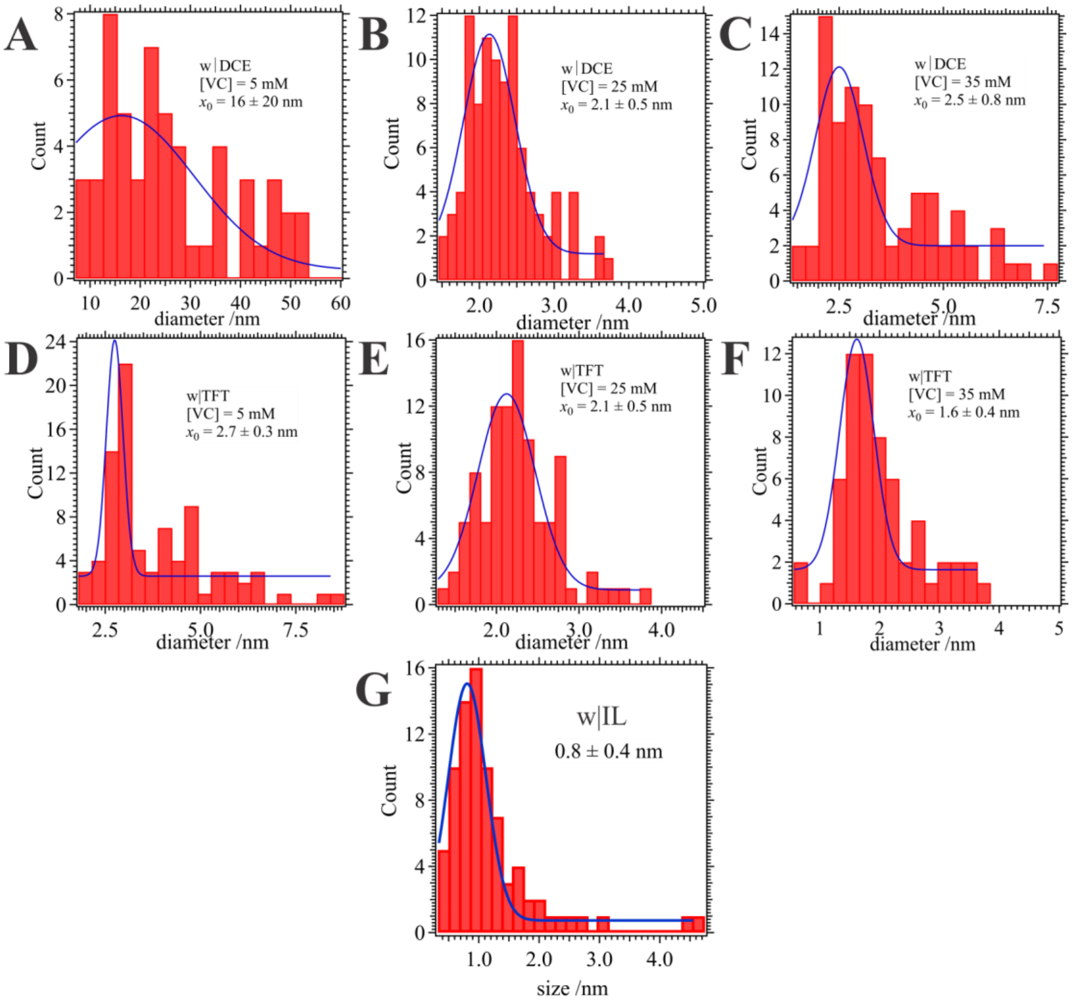

Figure S2: Histograms (bar plots) of Au NP sizes from TEM images shown in Figure 4 and 5 of the main text developed from micro w|DCE (**A-C**), w|TFT (**D-F**), and w|IL (**G**) voltammetric experiments using Cells 1 and 3 (see Scheme 2) with changing [VC] as indicated inset. For w|IL experiments see Figure 5 of the main text. Solid curves were generated form a Gaussian fitting in Igor Pro version 9.0.5, see also equation S1. Also inset are the final Gaussian peak fitting parameters for each experiment in which x_0 is the mean or peak value, while w is the width-at-half-maximum or 1σ.

General equation for the Gaussian fitting:

$$f(x) = A\exp\left(\frac{(x - x_0)^2}{w}\right) \quad [S1]$$

Where x_0 is the mean, A the preexponential fitting factor, and w the width which is equal to one standard deviation (σ).

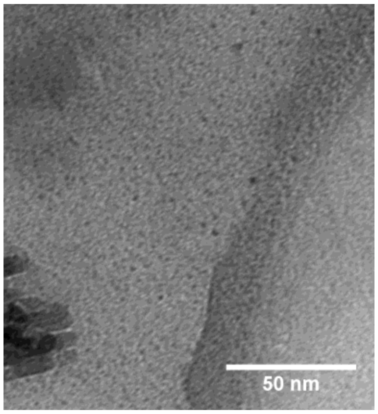

Figure S3: Higher magnification TEM micrograph of the one shown in Figure 5B of the main text.

3.0 Comsol simulation details and scanning electrochemical microscopy (SECM) results

The Comsol Multiphysics is a finite element analysis software in which a 2D axial symmetric geometry as shown in Figures S3B and S3C employing the 'Transport of Diluted Species' and 'Chemical Engineering' modules. For simplicity, the geometry was constructed out of a series of rectangles with an additional square added to the bottom of the geometry along the substrate boundary to facilitate greater control while defining the mesh.

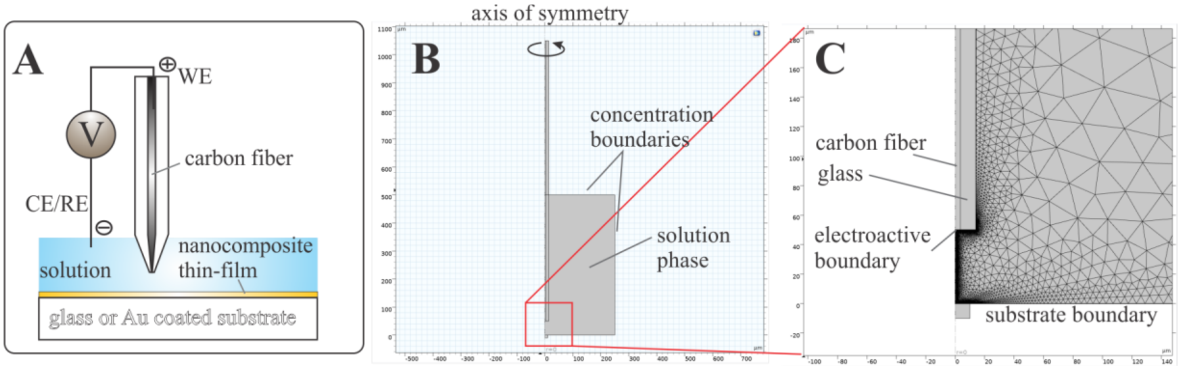

Figure S4: (**A**) Scheme of the ultramicroelectrode (UME) configuration for SECM. A low (**B**) and higher (**C**) magnification image of the 2-dimensional, axial symmetric SECM geometry built in Comsol Multiphysics with the domain and boundary conditions defined inset. C includes the triangular mesh with a tip-to-substrate distance (d) of 50 µm.

Diffusion was defined using Fick's laws as detailed below for a 2D axial symmetric/cylindrical space,

$$\frac{\partial c_{i^z}(r,\varphi,z,t)}{\partial t} = D_{i^z}\nabla^2 c_{i^z}(r,\varphi,z,t) \qquad \text{[S2]}$$

$$\nabla^2 = \frac{1}{r}\frac{\partial}{\partial r}\left(r\frac{\partial}{\partial r}\right) + \frac{1}{r^2}\left(\frac{\partial^2}{\partial \varphi^2}\right) + \left(\frac{\partial^2}{\partial z^2}\right) \qquad \text{[S3]}$$

While the Butler-Volmer kinetics described the oxidation/reduction (equation S4) of the redox mediator at the carbon fiber UME surface or 'electroactive boundary' as shown in Figure S3C. The rates of the forward (k_f) and reverse (k_b) reactions from equation S4 are thus provided through equations S5 and S6, respectively.

$$\mathrm{Fc} \underset{k_b}{\overset{k_f}{\rightleftharpoons}} \mathrm{Fc}^+ + e^- \qquad [S4]$$

$$k_f = k^o \exp\left[(1-\alpha) f\left(E_{appl.} - E^{o'}\right)\right] \qquad [S5]$$

$$k_b = k^o \exp\left[-\alpha f\left(E_{appl.} - E^{o'}\right)\right] \qquad [S6]$$

In this case, k° is the standard rate constant (1 m s^{-1}), α is the transfer coefficient (0.5), while f = nF/RT; whereby, n = 1 is the number of electrons transferred, F is Faraday's constant (96485.33 C mol^{-1}), R is the universal gas constant (8.314 J mol^{-1} K^{-1}), and T is the absolute temperature (298.15 K). $E_{appl.}$ and $E^{\circ\prime}$ are the applied potential and the standard redox potential, respectively. For simplicity, $E^{\circ\prime}$ was set equal to 0.25 V. E_{appl} was described either by a constant potential greater than $E^{\circ\prime}$ for probe approach curve simulations (*i.e.*, ~0.5 V) or as a triangular wave function[2] (equation S7) for cyclic voltammetric ones.

$$f(t) = E_i + \frac{2(E_f + E_i)}{\pi} \operatorname{asin}\left(\sin\left(\frac{\pi v t}{2(E_f + E_i)}\right)\right) \qquad [S7]$$

in which E_f, E_i, v, and t are the final potential, initial potential, scan rate, and time, respectively.

The current at the electrode boundary was calculated by,

$$I = nF \int D_{i^z} \nabla^2 c_{i^z}\left(r, \varphi, z, t\right) r dr d\varphi \qquad [S8]$$

Both time-dependent and stationary solvers were added to the model. The former was used to perform simulate cyclic voltammetric (CV) experiments in order to refine the triangular mesh. During mesh refinement, the tip was positioned at d = 50 µm and a CV was simulated and the steady state current ($i_{\mathrm{T},\infty}$) compared to the calculated value provided by equation S9,

$$i_{\mathrm{T},\infty} = 4nFc_i^* D_i r_a \qquad [S9]$$

such that c_i^* and D_i are the bulk concentration and diffusion coefficient of species i, while r_a is the radius of the UME electroactive disc. D_i was assumed to be 1.35×10^{-5} cm^2 s^{-1} for ferrocene methanol. Simulated CVs were performed the $i_{\mathrm{T},\infty}$ value compared to the calculated one from equation S9 and the mesh refined until it varied by <0.1%.

The substrate boundary (Figure S3C) was defined as 'insulating' for a non-conductive substrate and 'concentration' with a value of c_i^* for a conducting one, while for an intermediate dielectric surface the following two flux equations for Fc and Fc$^+$ were applied,

$$J_{Fc^+} = -kc_{0,Fc^+} \qquad [S10]$$

$$J_{Fc} = k\left(c_{Fc}^* - c_{0,Fc}\right) \qquad [S11]$$

Where $c_{0,i}$ is the concentration of species i at the substrate surface and k is the rate of redox mediator re-reduction.

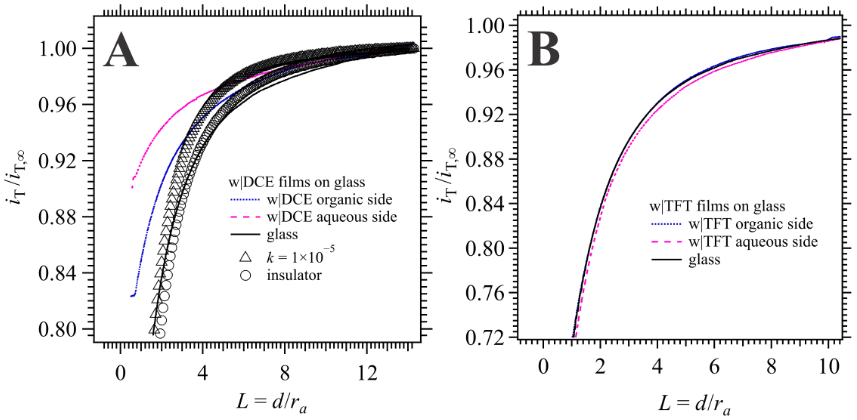

Figure S5: Solid traces are experimentally recorded probe approach curves (PACs) towards Au NP/poly(VC) films electrogenerated at a large (**A**) w|DCE or (**B**) w|TFT interface using a carbon fiber ultramicroelectrode (UME) with a radius of r_a = 3.5 µm and an $R_g = r_g/r_a \approx 5$; whereby, r_g is the radius of the outer glass sheath and r_a is the radius of the exposed carbon fiber disc. Marker traces are simulated curves based on the Comsol Multiphysics model described above for insulating and k values, as indicated inset.

4.0 Water contact angle measurements of as-prepared films at large-ITIES

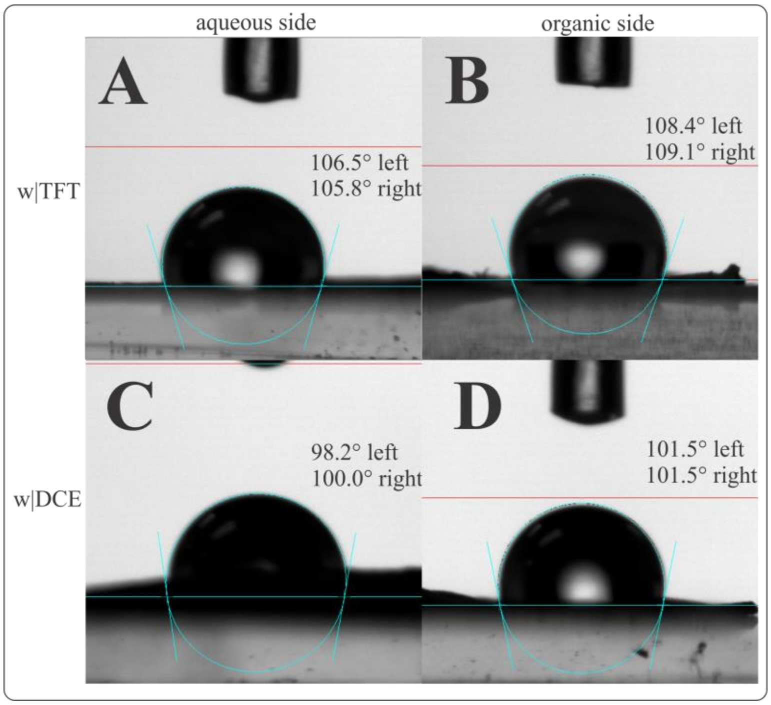

Figure S6: Images taken during water contact angle (WCA) measurements with on 2 µL water droplet dispensed onto the aqueous (**A** and **C**) or organic (**B** and **D**) sides of films formed at the large ITIES between w|TFT (**A** and **B**) or w|DCE (**C** and **D**) using Cell 2 after 25 voltammetric scans at 0.020 V s^{-1}; whereby, 20 mM of VC was added to the organic phase and 5 mM of $KAuCl_4$ to water. Measured angles have been indicated inset.

5.0 References

1. Olaya, A. J.; Ge, P.; Girault, H. H., *Electrochem. Commun.* **2012,** *19* (0), 101-104.
2. Rodgers, P. J.; Amemiya, S., *Anal. Chem.* **2007,** *79* (24), 9276-9285.
